# Using RNA Sequence and Structure for the Prediction of Riboswitch Aptamer: A Comprehensive Review of Available Software and Tools

**DOI:** 10.3389/fgene.2017.00231

**Published:** 2018-01-19

**Authors:** Deborah Antunes, Natasha A. N. Jorge, Ernesto R. Caffarena, Fabio Passetti

**Affiliations:** ^1^Scientific Computing Program (PROCC), Computational Biophysics and Molecular Modeling Group, Fundação Oswaldo Cruz, Rio de Janeiro, Brazil; ^2^Laboratory of Functional Genomics and Bioinformatics, Oswaldo Cruz Institute, Fundação Oswaldo Cruz, Rio de Janeiro, Brazil; ^3^Laboratory of Gene Expression Regulation, Carlos Chagas Institute, Fundação Oswaldo Cruz, Curitiba, Brazil

**Keywords:** riboswitch, RNA motif, riboswitch aptamer prediction, RNA secondary structure, RNA tertiary structure

## Abstract

RNA molecules are essential players in many fundamental biological processes. Prokaryotes and eukaryotes have distinct RNA classes with specific structural features and functional roles. Computational prediction of protein structures is a research field in which high confidence three-dimensional protein models can be proposed based on the sequence alignment between target and templates. However, to date, only a few approaches have been developed for the computational prediction of RNA structures. Similar to proteins, RNA structures may be altered due to the interaction with various ligands, including proteins, other RNAs, and metabolites. A riboswitch is a molecular mechanism, found in the three kingdoms of life, in which the RNA structure is modified by the binding of a metabolite. It can regulate multiple gene expression mechanisms, such as transcription, translation initiation, and mRNA splicing and processing. Due to their nature, these entities also act on the regulation of gene expression and detection of small metabolites and have the potential to helping in the discovery of new classes of antimicrobial agents. In this review, we describe software and web servers currently available for riboswitch aptamer identification and secondary and tertiary structure prediction, including applications.

## Introduction

Fifty years ago, the central dogma of molecular biology proposed a preferential flow of information, stating that DNA is transcribed into RNA, which in turn is translated into proteins with structural or catalytic functions (Crick, 1970; Albert et al., 2011). Since then, new findings have indicated that this theory was incomplete. For instance, in 2007, the ENCODE Project Consortium showed that, although most of the DNA is transcribed, only a fraction of the transcriptome is translated into proteins. RNA portions that do not encode proteins were then termed non-coding RNAs (ncRNA) (Crick, 1970; Mattick, 2001; Albert et al., 2011). Those ncRNAs belonging to the same class share precise sequence and structural characteristics, which have been conserved throughout several evolutionary processes. The degree of sequence conservation is smaller than that observed for protein-coding genes, but is crucial to explain the functional heterogeneity of the ncRNAs (Amaral et al., 2011; Qu and Adelson, 2012). One of the most significant examples of conserved functional RNAs are the riboswitches (Barrick and Breaker, 2007).

**Figure 1:**
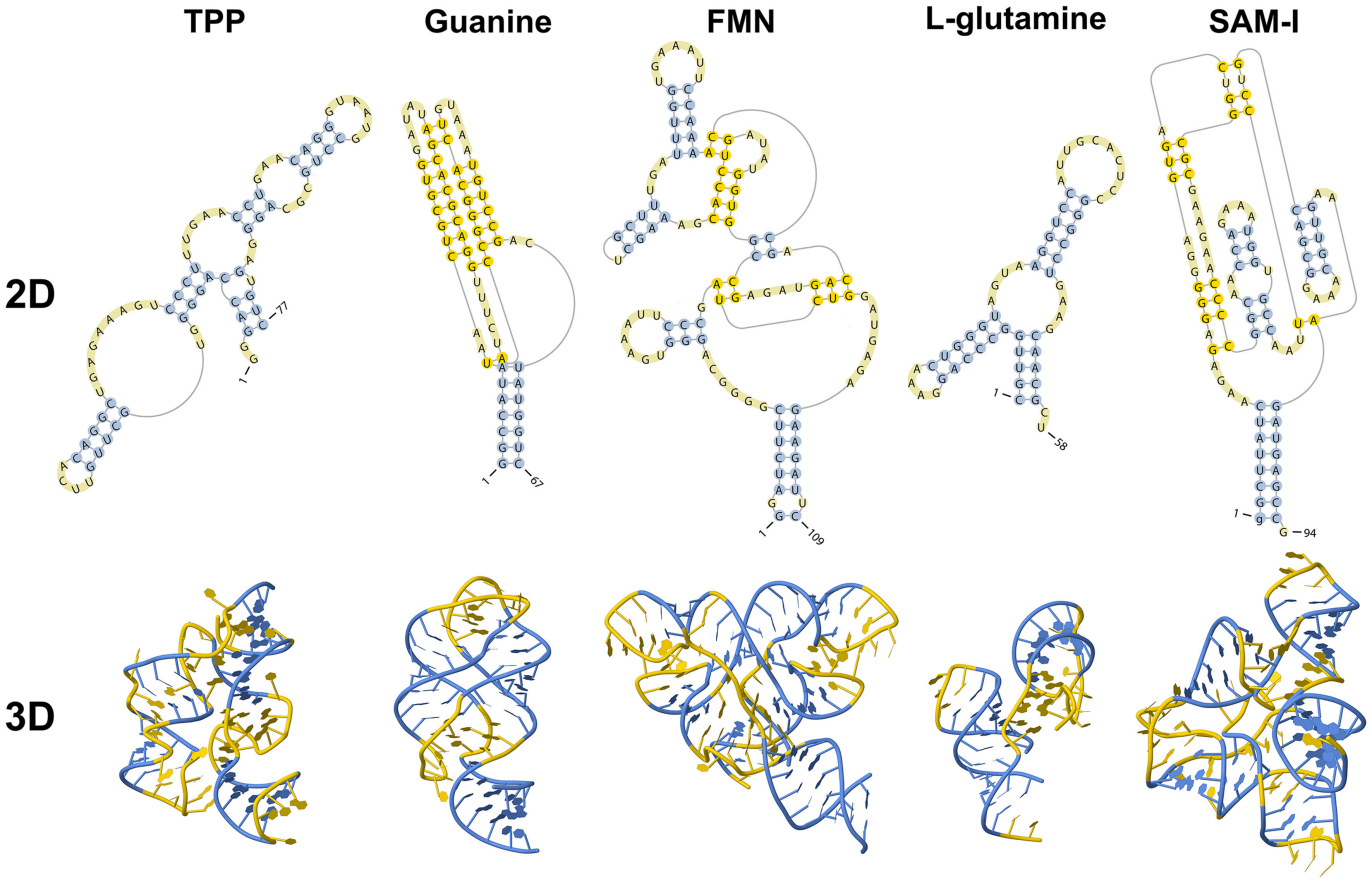
Secondary and tertiary structures of known riboswitches.

Riboswitches are natural RNA sensors located in the untranslated regions (UTRs) or the introns within an mRNA sequence. These sensors are capable of binding a great variety of small molecules, such as vitamins, amino acids, and nucleotides (Edwards and Batey, 2010; Breaker, 2012) and control the transcription or translation of the host mRNA. Riboswitches can be classified into different classes according to their binding metabolite, being the largest class the one including those capable of recognizing coenzymes, such as adenosylcobalamin (AdoCbl) (Vitreschak et al., 2003), thiamine pyrophosphate (TPP) (Winkler W. et al., 2002), flavin mononucleotide (FMN) (Winkler W. C. et al., 2002), *S*-adenosylmethionine (SAM) (Winkler et al., 2003), *S*-adenosylhomocysteine (SAH) (Wang et al., 2008), tetrahydrofolate (THF) (Ames et al., 2010) and molybdenum/tungsten cofactors (Moco/Tuco) (Regulski et al., 2008). Riboswitches belonging to the second largest group bind purines and some derivate purine compounds, such as adenine (Mandal and Breaker, 2004a), guanine (Batey et al., 2004), pre-queuosine-1 (preQ_1_) (Roth et al., 2007), deoxyguanosine (dG) (Wacker et al., 2011), cyclic-di-GMP (c-di-GMP) (Sudarsan et al., 2008), and cyclic-di-AMP (c-di-AMP) (Nelson et al., 2013). They also recognize amino acids, including lysine (Serganov et al., 2008), glycine (Mandal, 2004), and glutamine (Ames and Breaker, 2011). Other metabolites include metal cations such as Mg^2+^ (Cromie and Groisman, 2010), the halide anion F^−^ (Chawla et al., 2015) and glucosamine-6-phosphate (GlcN6P) (Klein, 2006). However, riboswitch classification could be larger as there are several putative structures yet to be validated and orphan riboswitches yet to be identified (reviewed by Peselis and Serganov, 2014). Examples of known riboswitches are depicted in Figure 1.

Genes regulated by riboswitches are involved in the biosynthesis, catabolism, signaling or transport of its binding metabolite, which creates a negative feedback regulatory mechanism to maintain the adequate levels of this molecule in metabolic processes (Mandal and Breaker, 2004b). When the levels of the metabolite increase, binding to the riboswitch occurs, leading to down regulation of the expression levels of the metabolite-related genes and, consequently, of the metabolite itself. This negative feedback mechanism can be considered as a fast reaction to changes in the environmental metabolite concentration that does not require the assistance of other supporting molecules (Serganov and Nudler, 2013), which consequently minimizes energy waste (Garst and Batey, 2009).

The structure of a riboswitch includes the aptamer and the expression platform, both of which are connected by the switching sequence. The aptamer region is evolutionarily conserved and responsible for metabolite recognition and binding (Tucker and Breaker, 2005; Hammann and Westhof, 2007; Serganov and Nudler, 2013). Binding of a metabolite induces a structural change in the expression platform, which is a highly variable region (Serganov and Nudler, 2013). This last modification controls gene expression (Garst and Batey, 2009). An example of this class of riboswitch is the guanine riboswitch, which is present in the *xpt-pbuX* operon of *Bacillus subtilis* (Ottink et al., 2007; Peselis and Serganov, 2014). In some riboswitches, such as the SAM-II riboswitch in the *metX* transcript of the Sargasso Sea metagenome, both aptamer and expression platform are merged into a single region (Coppins et al., 2007; Haller et al., 2011). In this particular case, SAM binding promotes the formation of a pseudoknot^1^ structure, which includes the Shine-Dalgarno sequence, preventing its recognition by the ribosome.

The “ON” and “OFF” states of riboswitches depend on metabolite binding (Garst et al., 2011). So far, the only known exception is the adenine riboswitch present in the *add* gene of the thermophile *Vibrio vulnificus*. In 2013, Reining et al. (2013) demonstrated the occurrence of three stable conformations for this riboswitch. In one of them, the metabolite was inside the structure and a free Shine-Dalgarno sequence allowed translation. In the two other conformations, the metabolite was not inside the riboswitch and the Shine-Dalgarno sequence was not free. The difference between these two ligand-free conformations is that one of them, which the authors termed apoB, cannot interact with the metabolite. To adjust its 3D-structure to the other ligand-free conformation able to bind adenine, termed apoA, a change in the environmental temperature and in metabolite concentration is needed.

The aptamer has an extremely high specificity to bind the metabolite, which allows it to act in the presence of many related compounds (Tucker and Breaker, 2005). For instance, the AdoCbl riboswitch cannot bind to methylcobalamin or cyanocobalamin (Nahvi, 2004), and the TPP-binding riboswitch does not interact with thiamine or thiamine monophosphate (TMP) (Lang et al., 2007). This specificity is due to the evolutionary conservation of sequence and structural features. If mutations occur within metabolite-binding regions, the function of the riboswitch can be affected or even abolished (Lai, 2003).

**Figure 2:**
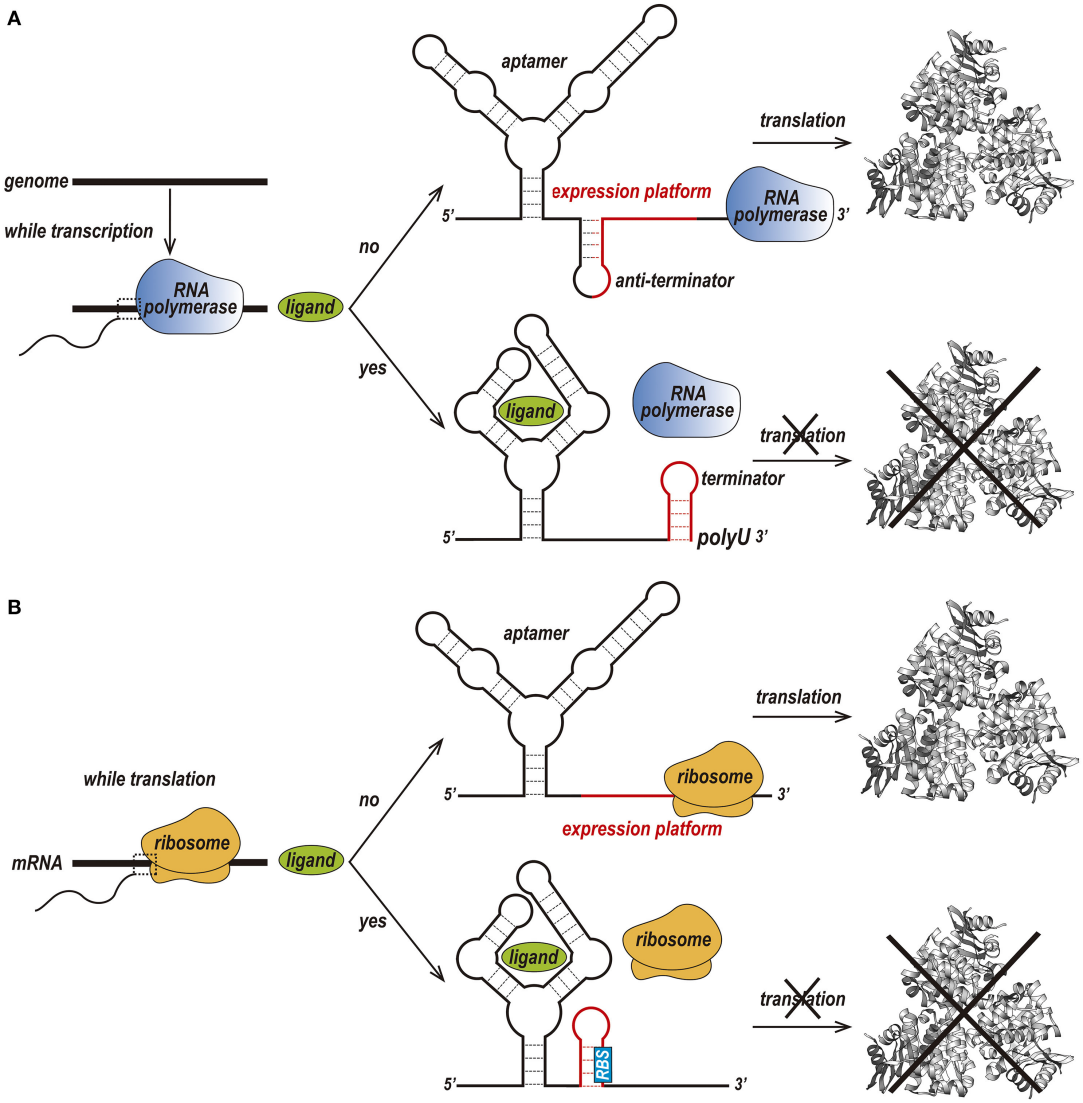
Two different forms of the riboswitch regulatory mechanism. **(A)** Premature termination of transcription. In the absence of a ligand, transcription of the downstream gene is permitted due to the formation of an anti-terminator stem. Upon binding of the ligand to the aptamer, a terminator stem is assembled instead the anti-terminator, and transcription in terminated. **(B)** Prevention of translation initiation. In the absence of a ligand, a ribosome binds to the ribosome-binding site (RBS) of an mRNA sequence and initiates translation. When the ligand is available, the RBS is sequestered and is not recognized by the ribosome, preventing translation to occur (Kim and Breaker, 2008).

Riboswitches can be found in the three kingdoms of life, *procaria, fungi* and *plantae* and can regulate transcription and translation in two different ways (Nudler and Mironov, 2004; Thore et al., 2006). In prokaryotes, riboswitches are usually located within the 5′ UTR region and act by prematurely terminating transcription (Figure 2A) or preventing the translation of its host mRNA (Figure 2B).

In premature termination of transcription, the structure of the expression platform folds, giving rise to either a terminator or an anti-terminator hairpin (Serganov and Nudler, 2013; Machtel et al., 2016). For instance, in the above-mentioned guanine binding riboswitch from the *Bacillus subtilis xpt-pbuX* operon, binding to guanine leads to the formation of a Rho-independent transcription terminator, while the ligand-free conformation forms an anti-terminator hairpin. The Mg^2+^ and FMN riboswitches, which are found in the *mgtA* transcript from *Salmonella enterica* serovar Typhimurium and the *ribB* transcript from *Escherichia coli*, respectively, prevent transcription elongation by a Rho-dependent transcription termination mechanism (Hollands et al., 2012). Upon riboswitch-metabolite binding, Rho binds to the transcribing mRNA, translocates up to the RNA–separates the transcribing mRNA from the template DNA thereby terminating transcription prematurely (Machtel et al., 2016).

Prevention of translation initiation occurs due to the absence of the ribosome-binding site (RBS) (Machtel et al., 2016). Examples of such riboswitches are the SAM-II riboswitch in the *metX* transcript of the Sargasso Sea metagenome (Gilbert et al., 2008), the adenine riboswitch within the *Add* mRNA from *Vibrio vulnificus* (Reining et al., 2013), and the lysine riboswitch in the *lysC* transcript from *E. coli*. In conditions of high lysine concentration, the expression platform of these riboswitches acquires a structure that simultaneously prevents translation and exposes RNase E cleavage sites (Peselis and Serganov, 2014).

In eukaryotes, only the TPP-binding riboswitch has been described where they may control splicing by either halting or promoting gene expression (Chen et al., 2011; Peselis and Serganov, 2014). In plants, such as *Arabidopsis thaliana, Oryza sativa*, and *Poa secunda* (Bocobza et al., 2007; Wachter et al., 2007), the 3′ UTR region of the *THIC* gene is highly conserved and harbors a TPP riboswitch. In this type of mRNAs, the start codon is followed by an intron, a small exon and a second intron that is tightly linked to the TPP riboswitch. This last intron may be kept or removed according to the intracellular TPP concentration. After binding to TPP, an alternative splice site is exposed, and the entire intron is removed along with its poly-adenylation site, thus generating an unstable transcript with several poly-adenylation sites (Bocobza and Aharoni, 2014).

In fungi such as *Aspergillus oryzae* (Kubodera et al., 2003) and *Neurospora crassa* (Li and Breaker, 2013), the TPP riboswitch is located within the 5′ UTR region. In this organism, when TPP levels are increased, metabolite binding to the riboswitch exposes an alternative splicing site while retaining part of its intron. This event changes the open reading frame and interrupts the biosynthesis of thiamine (Bocobza and Aharoni, 2008). The TPP riboswitch employs a similar mechanism in the transcription of the *THI4* and *THIC* genes from *algae* such as *Chlamydomonas reinhardtii* and *Volvox carteri* (Croft et al., 2007).

In 2009, Ray et al. published the discovery of an RNA switch structure in the 3′ UTR region of the human *VEGFA* gene (Ray et al., 2009). In conditions close to hypoxia, the structural conformation of the VEGF 3′UTR allows the interaction with the hnRNPl protein, which stabilizes and increases VEGFA translation. In normal oxygenation conditions, hnRNPl is degraded, and the VEGF mRNA binds to the GAIT complex, inhibiting translation. Different from riboswitches, the VEGF 3′UTR binds to two different protein elements to control gene expression. Nevertheless, the discovery of an RNA switch in human cells highlights the possibility of similar mechanisms playing essential roles in translation and transcription regulation in animal cells. Therefore, large-scale prediction of RNA motifs can serve as a tool to uncover these mechanisms and enhance our current knowledge of riboswitches and analog.

In the particular case of riboswitches, a single RNA sequence is capable of adopting, at least, two stable secondary structures in order to regulate the expression of a given gene. These structures are conserved throughout evolution in spite of sequence variations (Ritz et al., 2013). The information of the 3D aptamer structure became essential to investigate the mechanism of regulation of these switching functional ncRNAs. Structural information is necessary to characterize structural changes of riboswitch aptamers and fully to understand their role in the cell.

RNA structure is hierarchical, beginning with the linear ribonucleotide sequence, then a set of base-pairing interactions form the secondary structure, and sequentially the tertiary structure determines the spatial shape (Onoa and Tinoco, 2004). Like proteins, RNA motifs present structure-function relationships, and traditional experimental methods such as X-ray crystallography and nuclear magnetic resonance (NMR) provide critical insight into the details of this relation. However, these methods have limitations. In X-ray crystallography, due to the flexible nature of RNA molecules, it becomes difficult to grow crystals that can adopt unstructured components and multiple conformations. NMR experiments are limited to small RNAs (Ke and Doudna, 2004). In the Protein Data Bank (PDB) (Berman et al., 2000), only approximately 0.9% of all deposited structures correspond to RNA structures (accessed November 2017). The smaller number of RNA structures experimentally resolved makes it necessary to use computational methods to aid in determining the structures of RNAs.

Different computational tools were developed to search for novel riboswitches, this allows the identification of robust candidates prior to experimental validation. Several tools were also developed to predict RNA secondary structure, as well as 3D structure for RNAs. In this paper, we summarize currently available tools for riboswitch discovery and structure prediction. These tools are divided into three categories: prediction of RNA motif, prediction of RNA secondary structure, and prediction of RNA tertiary structure, according to their main method.

## Prediction of RNA motif

There are several methods for predicting RNA motifs, such as using an algorithm for predicting the secondary structure and then compare the conserved stem-loops (like RiboSW, Chang et al., 2009), searching for riboswitch specific sequence motives followed by the comparison of the secondary structures (riboswitch Finder, Bengert and Dandekar, 2004; RibEx, Abreu-Goodger and Merino, 2005; and DRD, Havill et al., 2014) and the usage of probabilistic models such as HMM and CM (HMMER, Mistry et al., 2013; Infernal, Nawrocki and Eddy, 2013b).

### HMMER

HMMER uses Hidden Markov Model (HMM) to create a position-specific score matrix based on primary sequence conservation (Krogh et al., 1994; Eddy, 1998; Mistry et al., 2013). Briefly, HMM is a statistical Markov model in which the modeled system is assumed to be a Markov chain with unobserved (hidden) states. nhmmer is a part of the HMMER and search nucleotide queries against a nucleotide sequence database (Wheeler and Eddy, 2013). The software can create a matrix with only one sequence, but its accuracy is improved when a larger set of reliable sequences is provided. A variety of input sequence file formats can be used as alignment file format (Stockholm, Aligned FASTA, Clustal, NCBI PSI-BLAST, PHYLIP, Selex, UCSC SAM A2M); and unalignment file (FASTA, EMBL, Genbank). The probabilistic HMM model searches for sequence homologs in an available sequence database, accounting for substitutions, insertions, and deletions. The program output ranks a list of the hits with the most significant matches to the query. Each hit represents a region of local similarity of the HMM to a subsequence of a full target database sequence. An alignment of the matched sequence to the model with the confidence value which each position is aligned is also shown. The current version of this software (Version 3.1) can be found at http://hmmer.org/.

### Infernal

Infernal (INFERence of RNA ALignment) was first created by Sean Eddy in 2002 (Eddy et al., 2002). The 1.1.2 version (July, 2016) (Nawrocki and Eddy, 2013b) is used by the Rfam database (Nawrocki et al., 2015) to infer a set of homologous sequences of an RNA family. Infernal's algorithm implements covariance models (CMs), a particular case of stochastic context-free grammar (SCFGs), to create a probabilistic model that accounts for RNA sequence and secondary structure conservation that can be used to search for a particular structural pattern in user-provided sequences (Eddy et al., 2002). Further reading about SCFG is available in Giegerich (2014). First, the software utilizes a set of reliable sequence alignments, along with a common secondary structure annotation (Stockholm format), to create the CM model specific for that target RNA family. Then, it uses a dynamic programming algorithm to find similar sequence and structural patterns in a set of target sequences. The alignment and a set of trustworthy RNA sequences can be found in the Rfam database (http://rfam.xfam.org/). Infernal has many functions that are based on analogous ones in HMMER, such as output formatting, which is very similar to the two software packages. Infernal is available at http://eddylab.org/infernal/.

### Riboswitch finder

Developed in 2004 by Bengert and Dandekar (2004), this web-based tool employs user-provided nucleotide sequence to infer putative riboswitches by searching for specific sequence motives and obtain its secondary structure. The algorithm was tested with a consensus set of 13 known *Bacillus subtilis*-like riboswitches sequences. To use the Riboswitch finder, merely give any RNA sequence up to three million base pairs of nucleotides. The tool provides information on the position of the putative riboswitch, the minimum free energy (MFE) and a putative secondary structure alignment. The secondary structure and MFE are calculated using the Vienna RNA package (Lorenz et al., 2011). Riboswitch finder is available at http://riboswitch.bioapps.biozentrum.uni-wuerzburg.de/server.html.

### RibEx

RibEx (Riboswitch Explore) (Abreu-Goodger and Merino, 2005) is a web server to detect riboswitches and riboswitch-like elements (RLEs). Among others, RibEx can detect the Gram-positive T-box and the PyrR protein binding site based on sequence motifs that are unique to each particular class. RibEx employs an algorithm capable of finding bacterial regulatory motifs, built exclusively on sequence conservation of regulatory regions associated with at least one cluster of orthologous groups of proteins that can be found in at least five non-redundant genomes. After submitting the target primary sequence of interest, up to 40,000 bases, the software provides a scheme of the open reading frame (ORF) with the regulatory element found. The sequence corresponding to this element can be addressed with the NCBI's BLAST tool. RibEx is currently available at http://132.248.32.45/cgi-bin/ribex.cgi.

### RiboSW

The RiboSW is a webserver (Chang et al., 2009) able to identify up to 12 classes of riboswitches based on structural conformations and sequence conservation. The authors used the sequence and secondary structure information of 12 riboswitches annotated in Rfam to recognize fundamental structure components and to create HMM models. After a user sequence query, the software seeks for the combination of structural elements corresponding to one of the twelve riboswitch classes, and performs a functional local detection using HMMER (Mistry et al., 2013). This tool provides a sequence with secondary structure annotation and graph, the MFE, the HMM e-value and the RNA Logo graph, which compares the provided sequence with the corresponding Rfam riboswitch family.

The authors stated that the performance of their method is comparable to that of the CM employed by Rfam, except for the AdoCbl and TPP riboswitches, as RiboSW was not able to detect all the members of these riboswitch families. They suggested that this flaw may be due to the high structural variation found within these families. The webserver is hosted at http://ribosw.mbc.nctu.edu.tw/.

### RNAConSLOpt

RNAConSLOpt is a program for predicting consensus stable local optimal structures for multiple sequence alignment of related RNAs (Li et al., 2012). The program also allows predicting alternate consensus structures for riboswitches elements in bacteria 5′ UTRs. *De novo* prediction, with no previous knowledge about sequences and structures of known riboswitches, is possible. To predict ncRNA, RNAConSLOpt incorporates information of free energies of structures, covariance and conservation signals into enumerating ConSLOpt stack configurations. The program output consisting of all probable consensus local optimal stack configurations and consensus stable local optimal stack configurations ranked according to both free energy and the associated minimal energy barrier. RNAConSLOpt is available for download at http://genome.ucf.edu/RNAConSLOpt/.

### DRD: denison riboswitch detector

Using a dynamic programming algorithm that considers mismatches, the Denison Riboswitch Detector (DRD) (Havill et al., 2014) web server predicts up to 13 classes of riboswitches from DNA sequences. The applied algorithm breaks the query sequence into overlapping smaller sequences and searches for exclusive motifs belonging to each searched riboswitch class. Afterwards, the secondary structure is predicted using Mfold (Zuker, 2000) and is subsequently aligned with a consensus sequence of the riboswitch class. The query provides the position of the putative riboswitch along with its optimal and suboptimal secondary structure annotation and graph. The authors tested DRD using validated sequences annotated in Rfam. Overall, the software achieved 88–99% sensitivity and more than 99% specificity. The web server can be found at http://drd.denison.edu/.

### Riboswitch scanner

Riboswitch Scanner (Mukherjee and Sengupta, 2016) is a web server capable of detecting 24 riboswitch classes and identifying novel riboswitches. Its algorithm utilizes 5-fold cross-validated HMM models created by HMMER 3 (Mistry et al., 2013) to determine putative riboswitches. Through the submission of the nucleotide sequences in FASTA format, the server provides the position of the riboswitch, MFE, HMM score and E-value, and secondary structure annotation obtained by RNAfold (Lorenz et al., 2011). It is available at http://service.iiserkol.ac.in/~riboscan/application.html.

### Comparison among tools for riboswitch aptamer prediction based on RNA motif

**Figure 3:**
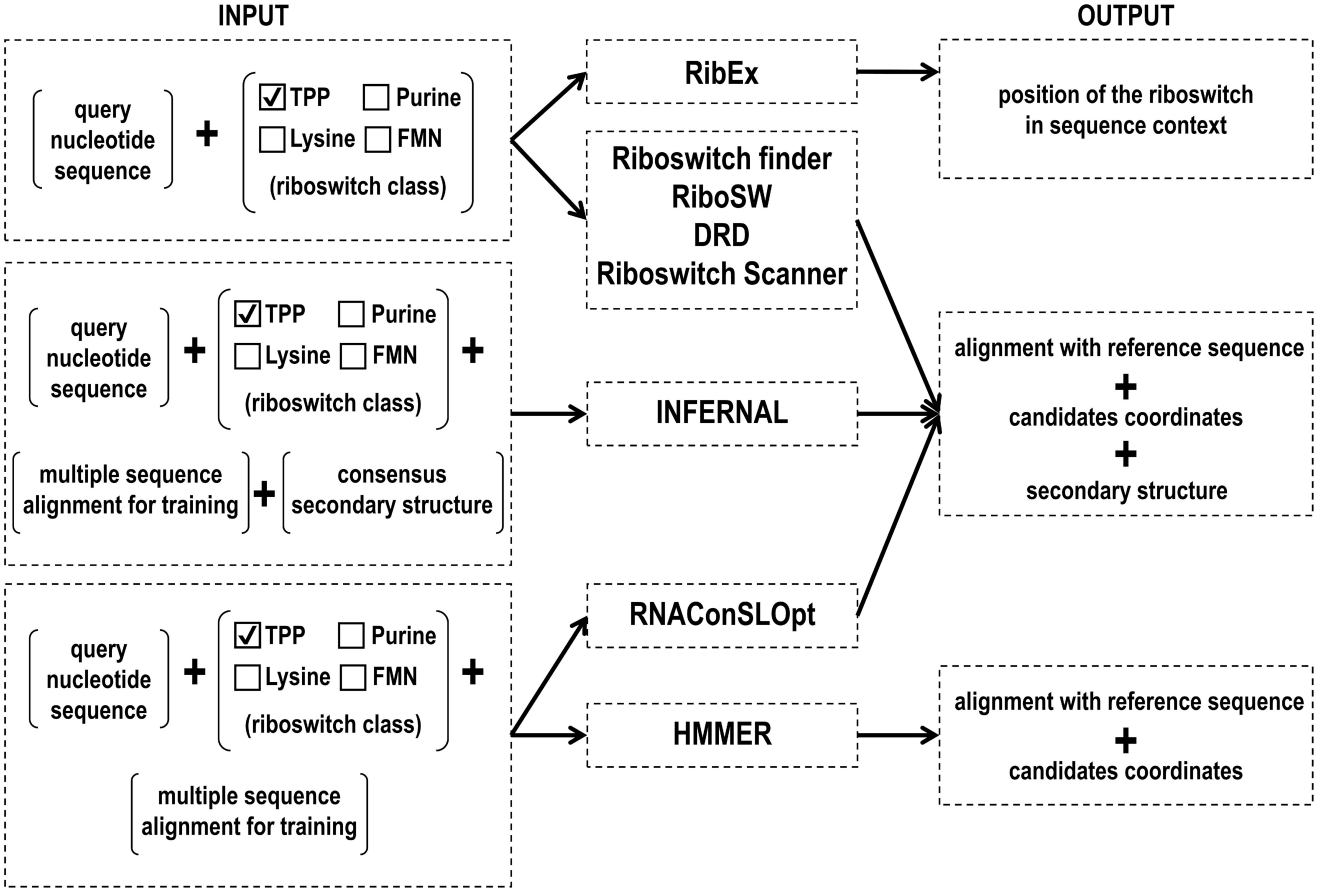
Input and output files of RNA motif prediction tools.

Riboswitches control the expression of genes involved in the biosynthesis and transport of ligands, as well as transcription factors (Mandal and Breaker, 2004b). Given their significant regulatory role in bacteria and in a few eukaryotic organisms, it is crucial to develop tools for the accurate identification of different riboswitch classes. Several approaches have been used for the computational identification of riboswitch aptamers (Figure 3, Table 1). The current riboswitch search tools employ hidden Markov model algorithm, covariance model, and machine learning methods, which often use riboswitch aptamers identified from seed alignments performed with sequences retrieved from the Rfam database.

**Table 1:** Feature comparison of softwares used for riboswitch identification.

**Feature**	**HMM**	**Infernal**	**Riboswitch Finder**	**RibEx**	**RiboSW**	**RNAConSLOpt**	**DRD**	**Riboswitch Scanner**
Considers structural conformations	No	Yes	Yes	No	Yes	Yes	Yes	Yes
Considers conserved functional sequences	Yes	No	Yes	Yes	Yes	Yes	Yes	No
Software package	Yes	Yes	No	No	Yes	Yes	No	No
Max input length	None	2 kb	3 Mb	40 kb	10 kb	None	None	None
# riboswitches	Any	Any	13	17+	12	Any	13	24
New user definition	Yes	Yes	No	No	Yes	Yes	Yes	Yes

Most of the tools described here are web-based. These instruments often impose restraints on the input sequence length and number of riboswitches that can be detected at once. They also rely on sequence or structural conservation of the aptamer to perform that analysis. Therefore, the aptamer prediction affects the detection of more variable riboswitches, such as the TPP and the Cobalamin, or smaller ones, such as the guanine riboswitch.

Several computational methods have been created to identify novel riboswitches and to characterize those that are already known. Amongst the methods that use primary sequences, the HMMER and Infernal tools stand out due to their ability to run locally, with the advantage of not having upload limits. Both methods utilize similar approaches by applying probabilistic models to sequence datasets to infer patterns.

DRD group (Havill et al., 2014) compared their server with RiboSW. The advantage of DRD compared to the other server is the ability to scan genome-scale files for riboswitches. In analyses of overall sequences obtained higher sensitivity (0.95) than RiboSW (0.85). DRD server was able to detect 64 instances that RiboSW was not identified, and 12 instances in which the opposite was true.

In 2009, Singh and collaborators compared the performance of HMM with other two CM web-based tools (Riboswitch finder and RibEx) in the search for ten riboswitches families on Rfam or RefSeq databases (Singh et al., 2009). Their results showed that HMM models run faster than CM and were more accurate than Riboswitch Finder and RibEx. The recently released version 1.1 of Infernal (Nawrocki and Eddy, 2013b) is reported to be 100 times faster than earlier versions and has been used for the identification of functional RNA homologs in metagenomic data (Nawrocki and Eddy, 2013a).

Although we agree that HMMER is faster than Infernal, our experience in searching for riboswitches in genomes does not corroborate their report of similar searches. In our case, most of the candidate regions found while using HMMER were not identified by Infernal, and the ones that were in common were discarded according to the following threshold filters: positive values for HMMER and values above the Rfam gathering score for Infernal, or *E*-values greater than 0.01 in both cases (unpublished data).

In a recent review on the computational prediction of riboswitches (Clote, 2015), Infernal was considered the most valuable tool to predict riboswitch aptamers mainly because the relevant Rfam database relies on Infernal for maintenance and extension. Some studies have used Infernal to identify riboswitches and other ncRNAs in archaeal metagenomes (Nawrocki and Eddy, 2013a; Gupta and Swati, 2016), species in the phyla *Actinobacteria* (Kang et al., 2017) and Proteobacteria (Leyn et al., 2014), *Methanobrevibacter ruminantium* (Nawrocki, 2014), *Neisseria gonorrheae* (Remmele et al., 2014), and *Brassica rapa* (Pang et al., 2015). Based on our knowledge, we also recommend the use of the Infernal program because this tool takes into account secondary structure conservation.

## Prediction of two- and three-dimensional RNA structures

### Prediction of RNA secondary structure

Most functional ncRNAs have secondary structures that are strictly related to their functions and that have been conserved during evolution. RNA secondary structures are defined by the arrangement of a set of base pairs non-covalently bound through hydrogen bonds, and can be considered a substructure of the global 3D structure. As it is difficult to obtain the experimental elucidation of RNA 3D structures and these structures are hierarchically folded, the computational prediction of RNA secondary structures provides key information to clarify the potential functions of RNAs. So far, a large number of computational studies have been carried out in the field of RNA secondary structure prediction. Prediction methods can be classified into two groups: single sequence analysis and multiple sequence analysis. Single sequence analysis is a traditional approach that consists of finding the structure with minimum free energy (MFE) of a single RNA sequence. On the other hand, the multiple sequence analysis has the advantage of providing higher prediction accuracy compared to single sequence analysis because it incorporates evolutionary information. Nonetheless, this approach is not always suitable, given that knowledge of a set of homologous sequences is required. Below, we list a few programs that perform these analyses.

#### UNAFold/Mfold

The Mfold software for RNA folding was published as a stand-alone option (Zuker, 1989). The first version of the Mfold package applied free energy minimization and the methodology was described by Freier et al. (1986). Following versions (2.1 to 2.3) used the parameters from Walter et al. (1994) and only in the recent version 3.6, free energy data from Mathews et al. (1999b) was incorporated into the algorithm. The Mfold web server was first created in 1995 (Zuker, 2003) and currently merged with DINAMelt (Markham and Zuker, 2005) (web server simulates hybridization and melting prediction of one or two single-stranded nucleic acids in solution) giving rise to UNAFold (Markham and Zuker, 2008). RNA folding prediction is available at http://unafold.rna.albany.edu/?q=mfold/rna-folding-form.

#### RNAfold

The RNAfold program belongs to the Vienna RNA package (Hofacker et al., 1994; Lorenz et al., 2011). RNAfold predicts the most thermodynamically stable structure compatible with a single RNA sequence using the standard algorithm of Zuker and Stiegler (1981). The prediction algorithm is based on dynamic programming. It finds a minimum free energy conformation using published values of stacking and destabilizing energies. Additionally, it can produce the base-pairing probability matrix via John McCaskill's partition function algorithm (McCaskill, 1990), which is a different O(n^3^) time dynamic programming algorithm. This methodology allows computation of the partition function of a nucleotide sequence over all possible unpseudoknotted secondary structures. RNAfold is available as a free software Vienna RNA package as well as web server (Hofacker, 2003) at http://rna.tbi.univie.ac.at/cgi-bin/RNAWebSuite/RNAfold.cgi.

#### RNAstructure

RNAstructure (Mathews et al., 1998; Reuter and Mathews, 2010) used to include a method to predict the lowest free energy structure. This tool could also provide a group of low free energy structures (Steger et al., 1984; Zuker, 1989). After it was expanded to foresee binding affinity of oligonucleotides to a complementary RNA target with OligoWalk (Mathews et al., 1999a,b; Lu and Mathews, 2008), a tool called Dynalign (Mathews and Turner, 2002; Uzilov et al., 2006; Harmanci et al., 2007) was added to enhance the accuracy of structural prediction by combining free energy minimization and comparative sequence analysis, and to obtain a low free energy structure common to two sequences with no obligation of sequence identity.

This tool incorporates additional data to drive the prediction of secondary structure such as enzymatic data (Mathews et al., 1999b), chemical mapping data (Mathews et al., 2004), SHAPE (Rice et al., 2014), and NMR data (Hart et al., 2008). Recent extensions include PARTS (Lu and Mathews, 2008), which calculates partition functions for secondary structures common to two sequences and can also produce a stochastic sampling of common structures (Mathews et al., 1999a). MaxExpect generates a very specific group of structures from a sequence of either RNA or DNA, and predicts the maximum expected accuracy structure, that is, a structure that maximizes pair probabilities (Lu et al., 2009). RNAstructure is an open-source program and can be found at http://rna.urmc.rochester.edu/RNAstructureWeb/ (Bellaousov et al., 2013).

#### SFold/Srna

Sfold is a nucleic acid folding and design software package accessible to the scientific community through web servers since 2003 (Ding et al., 2004). Sfold package currently consists of six modules: (i) Sirna, which provides computational tools for target accessibility prediction; (ii) Soligo, used for rational design of siRNAs; (iii) Sribo, used to predict antisense oligos and trans-cleaving ribozymes; (iv) STarMir, for CLIP-based prediction of microRNA binding sites and; (v) STarMirDB, a database of microRNA binding sites; (vi) Srna, which provides general statistical folding features. Srna implements tools and sampling statistics to analyze the Boltzmann ensemble of RNA secondary structures. All Sfold modules are available at http://sfold.wadsworth.org/cgi-bin/index.pl.

#### RNAalifold

RNAalifold predicts a consensus secondary structure for a set of previously aligned homologous RNA sequences. This approach is inherently limited by the quality of the input alignments. The first RNAalifold approach combines energy minimization with a simple scoring model to assess evolutionary conservation (Hofacker et al., 2002). Both an energy minimization algorithm and a partition function version are implemented in the Vienna RNA package (Lorenz et al., 2011). RNAalifold (Bernhart et al., 2008) was later optimized by incorporating a more accurate treatment of gaps and an elaborated model for the evaluation of sequence covariations resembling the RIBOSUM matrices (Klein and Eddy, 2003). Current limits are 3,000 nt and 300 sequences for an alignment and the program can be found at http://rna.tbi.univie.ac.at/cgi-bin/RNAWebSuite/RNAalifold.cgi.

#### LocARNA

LocARNA is a tool for RNA sequence multiple alignments (Will et al., 2007, 2012; Smith et al., 2010). The LocARNA multiple alignments are shown in conjunction with the predicted structure by RNAalifold (Bernhart et al., 2008). LocARNA computes pairwise alignments by dynamic programming using a progressive alignment strategy. LocARNA is part of the Freiburg RNA tools web server (Smith et al., 2010). LocARNA only needs RNA sequences as an input and simultaneously performs folding and alignment of the sequences. Specifications of other constraints or fixed input structures are also possible. Current limits are 2,500 nt for the longest sequence. The server is available at http://rna.informatik.uni-freiburg.de/LocARNA/Input.jsp.

#### IPknot

IPknot is a method for Integer Programming (IP)-based prediction of RNA secondary structures with a broad class of pseudoknots (Sato et al., 2011; Kato et al., 2012). The input data can be either a single RNA sequence or an alignment of RNA sequences. IPknot accepts maximum length sequences of 1500 nt and allows multiple alignments of RNA sequences in ClustalW format or multiple FASTA formats to predict their consensus secondary structure. IPknot is available at http://rtips.dna.bio.keio.ac.jp/ipknot/.

### Prediction of RNA tertiary structure

Similar to what is observed in proteins, the functions of an RNA molecule depend on its structure and dynamics, which are determined by its nucleotide sequence. The number of computational methods and algorithms to predict the 3D structure of proteins from its amino acid sequence is vast. Unfortunately, only a few are available for the prediction of RNA structure (Chojnowski et al., 2014).

To determine a 3D configuration with the best possible accuracy, knowledge-based approaches are the most suitable. Comparative (or homology-) modeling, for instance, which is based on sequence similarity, works properly when there is an experimentally elucidated structure to be used as a template (Baker and Sali, 2001). However, RNA templates are rarely available.

Physics-based approaches are successful for the prediction of relatively small molecules. These tools are comparatively more appropriate for building models of RNA molecules with less than ~40 nt and display reasonable reliability for molecules up to ~80 nt. The prediction of larger molecules is possible, but the reliability of the model decreases as the length of the sequence increases (Magnus et al., 2014).

The combination of knowledge- and physics-based approaches resulted in the development of the so-called *de novo* folding methods, which is the assembly of the target structure from small fragments derived from other known structures (Bujnicki, 2006). Here, we compiled some programs using different approaches to predict RNA 3D modeling.

#### MC-fold|MC-Sym

MC-Sym provides tertiary structures using the MC-Fold's secondary structures (Parisien and Major, 2008). The RNA-structure-prediction method is based on Nucleotide Cyclic Motifs (NCM), in which all nucleotides in fragments are circularly connected by covalent, stacking or pairing interactions. NCM property provides enough base-pairing context information to derive an efficient scoring function and allows the use of the same algorithm to predict both secondary and tertiary structures. MC-Sym exhaustively or probabilistically explores the conformational search space of an RNA molecule and produces structures satisfying secondary structure constraints. MC-fold|MC-Sym is available at http://www.major.iric.ca/MC-Fold/.

#### iFoldRNA

The iFoldRNA web server performs automated prediction of RNA structure and analyses of thermodynamic folding. In its previous version (Sharma et al., 2008), only prediction of short RNA molecules (<50 nt) was conceivable. The current version allows prediction of a few hundred nucleotides (Krokhotin et al., 2015). iFoldRNA also enables the automatic inclusion of two categories of constraints: base-pairing and nucleotide solvent accessibility. The prediction of 3D RNA structures is performed using a coarse-grained 3-bead RNA model (phosphate, sugar, nucleobase). Simulations are carried out using the Discrete Molecular Dynamics (DMD) simulation engine (Ding et al., 2008). A set of RNA molecules at different temperatures undergoes replica exchange to enhance conformation sampling. The server is available to the academic community at http://redshift.med.unc.edu/ifoldrna/.

#### ModeRNA

ModeRNA (Rother et al., 2011) builds models using the atomic coordinates of a known RNA molecule (template) and the alignment between the target and template sequences. The program interprets the sequence alignment as a set of instructions and uses it to build a model structure by copying the template structure, with the subsequent introduction of the variable parts. ModeRNA can model post-transcriptionally modified nucleosides and offers many functions to analyze and manipulate RNA structures, such as cleaning structures, analyzing geometry and obtaining the secondary structure. The latest version (1.7.1) can be accessed online at http://iimcb.genesilico.pl/modernaserver/.

#### MacroMoleculeBuilder (MMB; previously RNABuilder)

MMB (Flores et al., 2011) is a modeling program based on the fulfillment of constraints and restrictions applied to the template to build the models. It uses internal coordinates to calculate distances. It also considers base pairings, base stacking and torsion angles, and interatomic distances with aligned regions of the template structure to use them as restraints to model the target sequence. Then, it performs a Monte Carlo (MC) simulation of an unfolded RNA chain or preliminary model. MMB works with protein, DNA and RNA, and is available at https://simtk.org/projects/rnatoolbox.

#### FARNA/FARFAR

FARNA/FARFAR builds *de novo* models of small RNA motifs using fragments (1–3 nucleotides long) from existing RNA structures whose sequences match subsequences of the target RNA. The Fragment Assembly of RNA (FARNA) (Das and Baker, 2007) algorithm is a MC process for low-resolution conformational sampling. Combined with the FARNA protocol, the method for Fragment Assembly of RNA with Full Atom Refinement (FARFAR) (Das et al., 2010) optimizes the models using the physically realistic full-atom Rosetta energy function. FARNA/FARFAR protocol is available for sequences up to 32 nt at the Rosetta Online Server That Includes Everyone (ROSIE) (Lyskov et al., 2013) (http://rosie.rosettacommons.org/rna_denovo).

#### Vfold model

The Vfold model (Cao and Chen, 2011) is based on a multi-scaling strategy to predict RNA free energy landscapes and 3D structures based on an input sequence. The secondary structure is predicted from the nucleotide sequence, and the free energy landscape is employed to build the ensemble of 2D structures with the identification of the lowest free energy state. Thus, a 3D coarse-grained structure is constructed as a scaffold, based on the PDB-based fragment to find the lowest free energy state. Then, all atoms are added to the coarse-grained scaffold. Lastly, AMBER energy minimization is carried out to compute the final atomistic 3D structure. The web server (Xu et al., 2014) and the source codes are freely accessible at http://rna.physics.missouri.edu/.

#### RNAComposer

RNAComposer is a server for the prediction of the 3D structure in RNA molecules of up to 500 nt (Popenda et al., 2012; Biesiada et al., 2016). The server predicts RNA structure based on the secondary structure in dot- bracket notation provided by the user. It also allows the incorporation of distance restraints derived from the experimental data to strengthen the 3D predictions. The secondary structure is divided into fragments containing overlapping canonical base pairs to build the model. The fragments are related to 3D elements found in RNA FRABASE database (Popenda et al., 2008, 2010), which is a dictionary containing RNA 3D structure elements derived from structures deposited in the RCSB PDB. RNAComposer automatically assembles the 3D elements using overlapping canonical base pairs followed by the energy minimization in the torsion angle space and subsequently in the Cartesian atom coordinate space. For the construction of models, jobs may be submitted to http://rnacomposer.cs.put.poznan.pl/.

#### 3dRNA

The 3dRNA (Zhao et al., 2012; Wang et al., 2015) is an automated program that provides larger RNA models and complex topologies based on secondary structures. The 3D structure is constructed from smallest secondary elements (SSEs) in two steps: the assembly of SSEs into duplexes or hairpins, and then into whole structures, as the 3D structures of hairpins and duplexes can typically be created with high accuracy. 3dRNA can be found at http://biophy.hust.edu.cn/3dRNA.

#### SimRNA

SimRNA (Boniecki et al., 2015) is a computational method for RNA folding simulations and 3D structure prediction. This tool uses a coarse-grained representation of the nucleotide chain (three pseudoatoms per nucleotide) and a knowledge-based energy function and MC sampling scheme to produce moves in the 3D space with a statistical potential to estimate the free energy. Additionally, the available online program called SimRNAweb (Magnus et al., 2016) (http://genesilico.pl/SimRNAweb/submit) offers a user-friendly interface that permits the input of a sequence to fold RNA using *de novo* methods. Alternatively, the user can provide secondary structure and distance restraints and a 3D structure in the PDB format to jump-start the modeling close to the expected final outcome.

### Comparison among tools for riboswitch aptamer prediction and candidate evaluation based on RNA structure models

Riboswitches undergo conformational changes upon ligand binding and act as a switch at the transcriptional or translational levels. Given that riboswitches are functional entities that can undergo conformational changes, knowing their structures is of essential importance to understand the molecular mechanisms associated with their regulatory functions. Hence, predicting the structure of riboswitches can provide useful insight into the mechanism through which small molecules bind to RNAs, as well as shed light on how this process induces conformational changes in riboswitches.

The application of energy minimization methods for secondary structure prediction of the riboswitch expression platform domain is still limited as it involves conformational change. However, the prediction of this domain may be useful to support experimental assays. Barash and Gabdank (2010) predicted a single point mutation positioned in the non-conserved TPP riboswitch region responsible for transforming the terminator to an anti-terminator state.

The recent developments in the secondary structure prediction allow to include probing data, like SHAPE and DMS, for restriction and prediction of a structure with high accuracy (reviewed in Sloma and Mathews, 2015). Among the programs listed by us, the RNAstructure includes an option of incorporating the probing data as restraints.

Prediction of a single RNA sequence is still limited, especially when long RNA sequences (reviewed in Hamada, 2015) are involved. Comparative approaches using homologous sequence information increase the accuracy of as secondary structure prediction. In many circumstances, homologous RNA sequences of the target RNA sequence can be obtained, and it would be of interest to know the common secondary structure to those sequences (Gardner and Giegerich, 2004).

The common secondary structure is a fundamental element in riboswitch aptamers prediction. Programs such as Infernal, Riboswitch Finder, RiboSW, DRD, and Riboswitch Scanner use structural conformations for homologous searching. Secondary structure information is also crucial for tertiary structure prediction. In template-based methods, it assists in modeling mutations or structural changes, whereas in *de novo* methods, it allows for base pair constraints when creating 3D models. For instance, the MC-sym tool was used to construct models of the SAM-I riboswitch RNA segment by incorporating elements of the expression platform, and allowing the formation of an antiterminator (AT) helix in the 3D structures (Huang et al., 2013).

**Figure 4:**
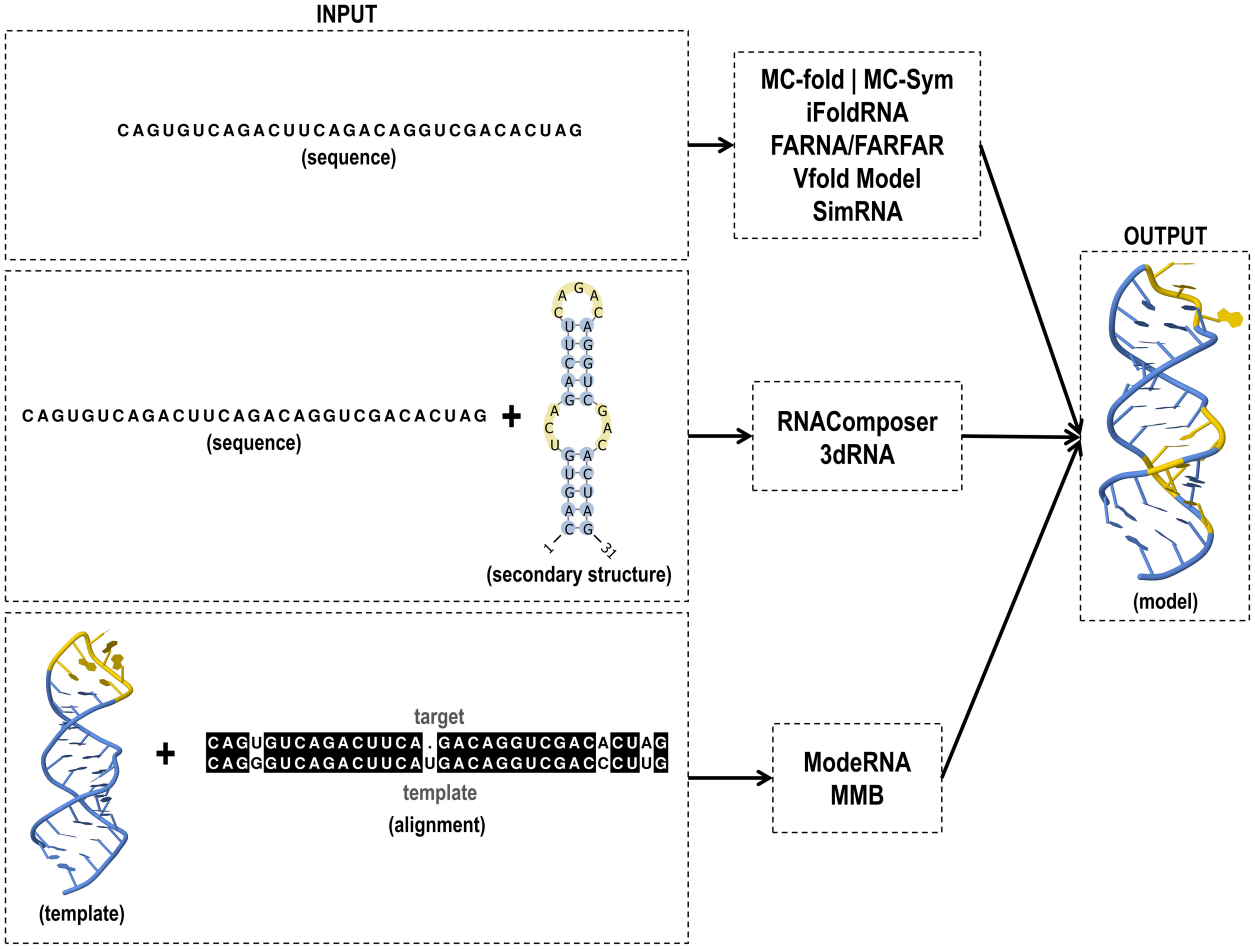
Input and output files of RNA tertiary structure prediction tools.

RNAComposer uses 2D restraint to create models and has provided positive results regarding the structural prediction of riboswitches. The server has been tested using a set often riboswitches containing pseudoknots and extensive tertiary interaction (Purzycka et al., 2015). In this set, nine examples were characterized with high accuracy and acceptable recovery of canonical and non-canonical base pairing and stacking. Input and output files of tertiary structure tools are shown in Figure 4.

Prompted by the increasing number of 3D RNA prediction framework methods, the RNA-Puzzles was started in 2012 (Cruz et al., 2012). RNA-Puzzles is a CASP-like (Moult et al., 2014) event in which collective blind experiments for the evaluation of 3D RNA structure prediction are carried out (Cruz et al., 2012; Miao et al., 2015). In the three rounds of RNA-Puzzles, predictions based on homology models already attained a high-level precision, providing useful insight to understand the RNA structure (Miao et al., 2017). Moreover, the prediction of ligand binding and subsequent conformational changes can also be described, but cannot be reliably guaranteed. Moreover, the prediction of ligand binding and subsequent conformational changes can also be described, but cannot be reliably guaranteed.

Gong et al. (2017) demonstrate in their review other approaches that aid the investigation of folding kinetics of aptamers and co-transcriptional folding kinetics using coarse-grained SOP model and, BarMap and helix-based computational approach, respectively. A new method StreAM-Tg (Jager et al., 2017) also allows analyzing structural transitions. This method gain insights into RNA dynamics based on a coarse-grained representation of RNA MD simulations.

Current modeling methods for template-based predictions have consistently reached a high accuracy level, i.e., now it is possible to model nearly all the structural details, provided that a reliable homologous structure is identified. Also, the ligand binding sites were readily inferred via homology (Miao et al., 2017). Different classes of riboswitches can be found in the RCSB PDB (Table 2), facilitating the use of model-based approaches such as ModeRNA and MMB.

**Table 2:** Classes of riboswitches that present experimentally resolved 3D structures.

**Class**	**No. of structures**	**PDB ID**	**Rfam accession**
AdoCbl-variant	4	4frg:b; 4frg:x; 4frn:a; 4frn:b	RF01689
c-di-GMP-I	17	3irw:r; 3iwn:a; 3iwn:b; 3mum:r; 3mur:r; 3mut:r; 3muv:r; 3mxh:r; 3ucu:r; 3ucz:r; 3ud3:r; 3ud4:r; 4yaz:r; 4yaz:a 4yb0:r; 4yb0:a; 4yb1:r	RF01051
c-di-GMP-II	2	3q3z:v; 3q3z:a	RF01786
Cobalamin	2	4gma:z; 4gxy:a	RF00174
FMN	6	3f2q:x; 3f2t:x; 3f2w:x; 3f2x:x; 3f2y:x; 3f30:x	RF00050
glmS	39	2gcs:b; 2gcv:b; 2h0s:b; 2h0w:b; 2h0x:b; 2h0z:b; 2ho6:b; 2ho7:b; 2nz4:p; 2nz4:q; 2nz4:r; 2nz4:s; 2z74:b; 2z75:b; 3b4a:b; 3b4b:b; 3b4c:b; 3g8s:p; 3g8s:q; 3g8s:r; 3g8s:s; 3g8t:p; 3g8t:q; 3g8t:r; 3g8t:s; 3g96:p; 3g96:q; 3g96:r; 3g96:s; 3g9c:p; 3g9c:q; 3g9c:r; 3g9c:s; 3l3c:p; 3l3c:q; 3l3c:r; 3l3c:s; 4meg:b; 4meh:b	RF00234
Glycine	19	3owi:a; 3owi:b; 3oww:a; 3oww:b; 3owz:a; 3owz:b; 3ox0:a; 3ox0:b; 3oxb:a; 3oxb:b; 3oxd:a; 3oxd:b; 3oxe:a; 3oxe:b; 3oxj:a; 3oxj:b; 3oxm:a; 3oxm:b; 3p49:a	RF00504
Lysine	16	3d0u:a; 3d0x:a; 3dig:x; 3dil:a; 3dim:a; 3dio:x; 3diq:a; 3dir:a; 3dis:a; 3dix:a; 3diy:a; 3diz:a; 3dj0:a; 3dj2:a; 4erj:a; 4erl:a	RF00168
MFR	16	3ski:a; 3ski:b; 3skl:a; 3skl:b; 3skr:a; 3skr:b; 3skt:a; 3skt:b; 3skw:a; 3skw:b; 3skz:a; 3skz:b; 3slm:a; 3slm:b; 3slq:a; 3slq:b	RF01510
preQ1-II	1	2miy:a	RF01054
PreQ1-III	1	4rzd:a	RF02680
Purine	40	1y26:x; 1y27:x; 2b57:a; 2ees:a; 2eet:a; 2eeu:a; 2eev:a; 2eew:a; 2g9c:a; 2xnw:a; 2xnz:a; 2xo0:a; 2xo1:a; 3ds7:a; 3ds7:b; 3fo4:a; 3fo6:a; 3g4m:a; 3gao:a; 3ger:a; 3ges:a; 3gog:a; 3got:a; 3la5:a; 3rkf:a; 3rkf:b; 3rkf:c; 3rkf:d; 4fe5:b; 4fej:b; 4fel:b; 4fen:b; 4feo:b; 4fep:b; 4lx5:a; 4lx6:a; 4tzx:x; 4tzy:x; 4xnr:x; 5c7u:b	RF00167
SAM	28	2gis:a; 2ydh:a; 2ygh:a; 3gx2:a; 3gx3:a; 3gx5:a; 3gx6:a; 3gx7:a; 3iqn:a; 3iqp:a; 3iqr:a; 3v7e:c; 3v7e:d; 4aob:a; 4b5r:a; 4kqy:a; 5fjc:a; 5fk1:a; 5fk2:a; 5fk3:a; 5fk4:a; 5fk5:a; 5fk6:a; 5fkd:a; 5fke:a; 5fkf:a; 5fkg:a; 5fkh:a	RF00162
SAM-I-IV-variant	2	4l81:a; 4oqu:a	RF01725
THF	10	3sd3:a; 3suh:x; 3sux:x; 3suy:x; 4lvv:a; 4lvw:a; 4lvx:a; 4lvy:a; 4lvz:a; 4lw0:a	RF01831
TPP	24	2cky:a; 2cky:b; 2gdi:x; 2gdi:y; 2hoj:a; 2hok:a; 2hol:a; 2hom:a; 2hoo:a; 2hop:a; 3d2g:a; 3d2g:b; 3d2v:a; 3d2v:b; 3d2x:a; 3d2x:b; 3k0j:e; 3k0j:f; 4nya:a; 4nya:b; 4nyb:a; 4nyc:a; 4nyd:a; 4nyg:a	RF00059
ydaO-yuaA	4	4qlm:a; 4qln:a; 4w90:c; 4w92:c	RF00379
ykoK	3	2qbz:x; 3pdr:x; 3pdr:a	RF00380

In the case of targets without sequence homology with previously experimentally resolved structures, modeling quality strongly depends on the size of the target. The third edition of RNA-Puzzles provided models for two small RNAs—the ZMP riboswitch (60-nt) and L-glutamine riboswitch (61-nt)—with approximately 6 Å of RMSD with the crystallographic structure. Although the tools are less accurate, they can correctly predict the overall global folding. Thus, the larger the targets without a template—ydaO riboswitch (108-nt)—, the less accurate will the predictions be (10 Å best-case RMSDs).

## Conclusion

In this review, we focused on the most frequently used software and web-based tools for riboswitch prediction that encompass RNA secondary and tertiary structures. Moreover, the study of riboswitches contributed to the analysis of computational methods used for the structural prediction of RNAs. The Rfam database, used by various RNA motif prediction tools, is maintained and extended by the Infernal software. Prediction of the secondary structure is useful not only for the functional analysis of RNAs but also to improve the search for structural RNAs in genomes and build 3D models. Although the prediction of single long sequences is still limited, comparative approaches like RNAalifold increase the prediction accuracy. For tertiary structure prediction, the availability of a homologous structure increases the quality of the predicted models. RNA-Puzzles experiments have shown that, in the absence a homologous structure, targets greater than 100-nt have less accurate models, although it is possible to predict the overall folding.

## Author contributions

DA and NJ: participated in the literature research, coordinated the structuring of the paper, and wrote the article; EC and FP: proposed and idealized this work, discussed topics, helped writing the article and supervised the organization of the whole process.

### Conflict of interest statement

The authors declare that the research was conducted in the absence of any commercial or financial relationships that could be construed as a potential conflict of interest.

## Footnotes

^1^Pseudoknot is an RNA structure in which the loop of a hairpin pairs with either a stem or a loop outside the original hairpin (Staple and Butcher, 2005).

## References

[B1] Abreu-Goodger C. Merino E. (2005). RibEx: a web server for locating riboswitches and other conserved bacterial regulatory elements. Nucleic Acids Res. 33, W690–W692. 10.1093/nar/gki445 15980564 PMC1160206

[B2] Albert B. Bray D. Hopkin K. (2011). Fundamentos da Biologia Celular. Porto Alegre: Artmed.

[B3] Amaral P. P. Clark M. B. Gascoigne D. K. Dinger M. E. Mattick J. S. (2011). lncRNAdb: a reference database for long noncoding RNAs. Nucleic Acids Res. 39, D146–D151. 10.1093/nar/gkq1138 21112873 PMC3013714

[B4] Ames T. D. Breaker R. R. (2011). Bacterial aptamers that selectively bind glutamine. RNA Biol. 8, 82–89. 10.4161/rna.8.1.13864 21282981 PMC3127080

[B5] Ames T. D. Rodionov D. A. Weinberg Z. Breaker R. R. (2010). A eubacterial riboswitch class that senses the coenzyme tetrahydrofolate. Chem. Biol. 17, 681–685. 10.1016/j.chembiol.2010.05.020 20659680 PMC3417113

[B6] Baker D. Sali A. (2001). Protein structure prediction and structural genomics. Science 294, 93–96. 10.1126/science.1065659 11588250

[B7] Barash D. Gabdank I. (2010). Energy minimization methods applied to riboswitches: a perspective and challenges. RNA Biol. 7, 90–97. 10.4161/rna.7.1.10657 20061789

[B8] Barrick J. E. Breaker R. R. (2007). The distributions, mechanisms, and structures of metabolite-binding riboswitches. Genome Biol. 8:R239. 10.1186/gb-2007-8-11-r239 17997835 PMC2258182

[B9] Batey R. T. Gilbert S. D. Montange R. K. (2004). Structure of a natural guanine-responsive riboswitch complexed with the metabolite hypoxanthine. Nature 432, 411–415. 10.1038/nature03037 15549109

[B10] Bellaousov S. Reuter J. S. Seetin M. G. Mathews D. H. (2013). RNAstructure: web servers for RNA secondary structure prediction and analysis. Nucleic Acids Res. 41, 471–474. 10.1093/nar/gkt290 23620284 PMC3692136

[B11] Bengert P. Dandekar T. (2004). Riboswitch finder–a tool for identification of riboswitch RNAs. Nucleic Acids Res. 32, W154–W159. 10.1093/nar/gkh352 15215370 PMC441490

[B12] Berman H. M. Westbrook J. Feng Z. Gilliland G. Bhat T. N. Weissig H. (2000). The protein data bank. Nucleic Acids Res. 28, 235–242. 10.1093/nar/28.1.235 10592235 PMC102472

[B13] Bernhart S. H. Hofacker I. L. Will S. Gruber A. R. Stadler P. F. (2008). RNAalifold: improved consensus structure prediction for RNA alignments. BMC Bioinformatics 9:474. 10.1186/1471-2105-9-474 19014431 PMC2621365

[B14] Biesiada M. Purzycka K. J. Szachniuk M. Blazewicz J. Adamiak R. W. (2016). Automated RNA 3D structure prediction with RNAComposer Methods Mol. Biol. 1490, 199–215. 10.1007/978-1-4939-6433-8_13 27665601

[B15] Bocobza S. E. Aharoni A. (2008). Switching the light on plant riboswitches. Trends Plant Sci. 13, 526–533. 10.1016/j.tplants.2008.07.004 18778966

[B16] Bocobza S. E. Aharoni A. (2014). Small molecules that interact with RNA: riboswitch-based gene control and its involvement in metabolic regulation in plants and algae. Plant J. 79, 693–703. 10.1111/tpj.12540 24773387

[B17] Bocobza S. Adato A. Mandel T. Shapira M. Nudler E. Aharoni A. (2007). Riboswitch-dependent gene regulation and its evolution in the plant kingdom. Genes Dev. 21, 2874–2879. 10.1101/gad.443907 18006684 PMC2049190

[B18] Boniecki M. J. Lach G. Dawson W. K. Tomala K. Lukasz P. Soltysinski T.. (2015). SimRNA: a coarse-grained method for RNA folding simulations and 3D structure prediction. Nucleic Acids Res. 44:e63. 10.1093/nar/gkv1479 26687716 PMC4838351

[B19] Breaker R. R. (2012). Riboswitches and the RNA world. Cold Spring Harb. Perspect. Biol. 4:a003566. 10.1101/cshperspect.a003566 21106649 PMC3281570

[B20] Bujnicki J. M. (2006). Protein-structure prediction by recombination of fragments. ChemBioChem 7, 19–27. 10.1002/cbic.200500235 16317788

[B21] Cao S. Chen S. J. (2011). Physics-based *de novo* prediction of RNA 3D structures. J. Phys. Chem. B 115, 4216–4226. 10.1021/jp112059y 21413701 PMC3072456

[B22] Chang T.-H. Huang H.-D. Wu L.-C. Yeh C.-T. Liu B.-J. Horng J.-T. (2009). Computational identification of riboswitches based on RNA conserved functional sequences and conformations. RNA 15, 1426–1430. 10.1261/rna.1623809 19460868 PMC2704089

[B23] Chawla M. Credendino R. Poater A. Oliva R. Cavallo L. (2015). Structural stability, acidity, and halide selectivity of the fluoride riboswitch recognition site. J. Am. Chem. Soc. 137, 299–306. 10.1021/ja510549b 25487435

[B24] Chen A. G. Y. Sudarsan N. Breaker R. R. (2011). Mechanism for gene control by a natural allosteric group I ribozyme. RNA 17, 1967–1972. 10.1261/rna.2757311 21960486 PMC3198590

[B25] Chojnowski G. Walen T. Bujnicki J. M. (2014). RNA bricks–a database of RNA 3D motifs and their interactions. Nucleic Acids Res. 42, D123–D131. 10.1093/nar/gkt1084 24220091 PMC3965019

[B26] Clote P. (2015). Computational prediction of riboswitches. Methods Enzymol. 553, 287–312. 10.1016/BS.MIE.2014.10.063 25726470

[B27] Coppins R. L. Hall K. B. Groisman E. A. (2007). The intricate world of riboswitches. Curr. Opin. Microbiol. 10, 176–181. 10.1016/j.mib.2007.03.006 17383225 PMC1894890

[B28] Crick F. (1970). Central dogma of molecular biology. Nature 227, 561–563. 10.1038/227561a0 4913914

[B29] Croft M. T. Moulin M. Webb M. E. Smith A. G. (2007). Thiamine biosynthesis in algae is regulated by riboswitches. Proc. Natl. Acad. Sci. U.S.A. 104, 20770–20775. 10.1073/pnas.0705786105 18093957 PMC2410077

[B30] Cromie M. J. Groisman E. A. (2010). Promoter and riboswitch control of the Mg^2+^ transporter MgtA from *Salmonella enterica*. J. Bacteriol. 192, 604–607. 10.1128/JB.01239-09 19897653 PMC2805325

[B31] Cruz J. A. Blanchet M. Boniecki M. Bujnicki J. M. Chen S. J. Cao S. (2012). RNA-Puzzles: a CASP-like evaluation of RNA three-dimensional structure prediction. RNA 18, 610–625. 10.1261/rna.031054.111 22361291 PMC3312550

[B32] Das R. Baker D. (2007). Automated *de novo* prediction of native-like RNA tertiary structures. Proc. Natl. Acad. Sci. U.S.A. 104, 14664–14669. 10.1073/pnas.0703836104 17726102 PMC1955458

[B33] Das R. Karanicolas J. Baker D. (2010). Atomic accuracy in predicting and designing noncanonical RNA structure. Nat. Methods 7, 291–294. 10.1038/nmeth.1433 20190761 PMC2854559

[B34] Ding F. Sharma S. Chalasani P. (2008). Ab initio RNA folding by discrete molecular dynamics: from structure prediction to folding mechanisms Ab initio RNA folding by discrete molecular dynamics: from structure prediction to folding mechanisms. RNA 14, 1164–1173. 10.1261/rna.894608 18456842 PMC2390798

[B35] Ding Y. Chan C. Y. Lawrence C. E. (2004). S fold web server for statistical folding and rational design of nucleic acids. Nucleic Acids Res. 32, 135–141. 10.1093/nar/gkh449 15215366 PMC441587

[B36] Eddy S. R. (1998). Profile hidden Markov models. Bioinformatics 14, 755–763. 10.1093/bioinformatics/14.9.755 9918945

[B37] Eddy S. R. Eddy S. Erdmann V. Barciszewska M. Symanski M. Hochberg A.. (2002). A memory-efficient dynamic programming algorithm for optimal alignment of a sequence to an RNA secondary structure. BMC Bioinformatics 3:18. 10.1186/1471-2105-3-18 12095421 PMC119854

[B38] Edwards A. L. Batey R. T. (2010). Riboswitches: a common RNA regulatory element. Nat. Educ. 3:9 Available online at: https://www.nature.com/scitable/topicpage/riboswitches-a-common-rna-regulatory-element-14262702

[B39] Flores S. C. Sherman M. A. Bruns C. M. Eastman P. Altman R. B. (2011). Fast flexible modeling of RNA structure using internal coordinates. IEEE/ACM Trans. Comput. Biol. Bioinform. 8, 1247–1257. 10.1109/TCBB.2010.104 21778523 PMC4428339

[B40] Freier S. M. Kierzek R. Jaeger J. A. Sugimoto N. Caruthers M. H. Neilson T.. (1986). Improved free-energy parameters for predictions of RNA duplex stability. Proc. Natl. Acad. Sci. U.S.A. 83, 9373–9377. 10.1073/pnas.83.24.9373 2432595 PMC387140

[B41] Gardner P. P. Giegerich R. (2004). A comprehensive comparison of comparative RNA structure prediction approaches. BMC Bioinformatics 5:140. 10.1186/1471-2105-5-140 15458580 PMC526219

[B42] Garst A. D. Batey R. T. (2009). A switch in time: detailing the life of a riboswitch. Biochim. Biophys. Acta 1789, 584–591. 10.1016/j.bbagrm.2009.06.004 19595806 PMC2783387

[B43] Garst A. D. Edwards A. L. Batey R. T. (2011). Riboswitches: structures and mechanisms. Cold Spring Harb. Perspect. Biol. 3:a003533. 10.1101/cshperspect.a003533 20943759 PMC3098680

[B44] Giegerich R. (2014). Introduction to stochastic context free grammars, in Methods in Molecular Biology, eds Gorodk J. Ruzzo W. L. (Totowa, NJ: Humana Press), 85–106. 10.1007/978-1-62703-709-9_5 24639156

[B45] Gilbert S. D. Rambo R. P. Van Tyne D. Batey R. T. (2008). Structure of the SAM-II riboswitch bound to S-adenosylmethionine. Nat. Struct. Mol. Biol. 15, 177–182. 10.1038/nsmb.1371 18204466

[B46] Gong S. Wang Y. Wang Z. Zhang W. (2017). Computational methods for modeling aptamers and designing riboswitches. Int. J. Mol. Sci. 18:2442. 10.3390/ijms18112442 29149090 PMC5713409

[B47] Gupta A. Swati D. (2016). Exploring riboswitches in archaeal metagenomes. J. RNAi Gene Silenc. 12, 536–543. Available online at: http://www.alliedacademies.org/journal-of-rnai-and-gene-silencing/exploring-riboswitches-in-archaeal-metagenomes.html

[B48] Haller A. Rieder U. Aigner M. Blanchard S. C. Micura R. (2011). Conformational capture of the SAM-II riboswitch. Nat. Chem. Biol. 7, 393–400. 10.1038/nchembio.562 21532598

[B49] Hamada M. (2015). RNA secondary structure prediction from multi-aligned sequences Methods Mol. Biol. 1269, 17–38. 10.1007/978-1-4939-2291-8_2 25577370

[B50] Hammann C. Westhof E. (2007). Searching genomes for ribozymes and riboswitches. Genome Biol. 8:210. 10.1186/gb-2007-8-4-210 17472738 PMC1895996

[B51] Harmanci A. O. Sharma G. Mathews D. H. (2007). Efficient pairwise RNA structure prediction using probabilistic alignment constraints in Dynalign. BMC Bioinformatics 8:130. 10.1186/1471-2105-8-130 17445273 PMC1868766

[B52] Hart J. M. Kennedy S. D. Mathews D. H. Turner D. H. (2008). NMR-assisted prediction of RNA secondary structure: identification of a probable pseudoknot in the coding region of an R2 retrotransposon. J. Am. Chem. Soc. 130, 10233–10239. 10.1021/ja8026696 18613678 PMC2646634

[B53] Havill J. T. Bhatiya C. Johnson S. M. Sheets J. D. Thompson J. S. (2014). A new approach for detecting riboswitches in DNA sequences. Bioinformatics 30, 3012–3019. 10.1093/bioinformatics/btu479 25015992 PMC4609007

[B54] Hofacker I. L. (2003). Vienna RNA secondary structure server. Nucleic Acids Res. 31, 3429–3431. 10.1093/nar/gkg599 12824340 PMC169005

[B55] Hofacker I. L. Fekete M. Stadler P. F. (2002). Secondary structure prediction for aligned RNA sequences. J. Mol. Biol. 319, 1059–1066. 10.1016/S0022-2836(02)00308-X 12079347

[B56] Hofacker I. L. Fontana W. Stadler P. F. Bonhoeffer L. S. Tacker M. Schuster P. (1994). Fast folding and comparison of RNA secondary structures. Monatshefte Chem. Chem. Mon. 125, 167–188. 10.1007/BF00818163

[B57] Hollands K. Proshkin S. Sklyarova S. Epshtein V. Mironov A. Nudler E.. (2012). Riboswitch control of Rho-dependent transcription termination. Proc. Natl. Acad. Sci. U S.A. 109, 5376–5381. 10.1073/pnas.1112211109 22431636 PMC3325659

[B58] Huang W. Kim J. Jha S. Aboul-ela F. (2013). The impact of a ligand binding on strand migration in the SAM-I riboswitch. PLoS Comput. Biol. 9:e1003069. 10.1371/journal.pcbi.1003069 23704854 PMC3656099

[B59] Jager S. Schiller B. Babel P. Blumenroth M. Strufe T. Hamacher K. (2017). StreAM-Tg: algorithms for analyzing coarse grained RNA dynamics based on Markov models of connectivity-graphs. Algorithms Mol. Biol. 12, 1748–7188. 10.1186/s13015-017-0105-0 PMC5450175 28572834

[B60] Kang I. Kim S. Islam M. R. Cho J.-C. (2017). The first complete genome sequences of the acI lineage, the most abundant freshwater Actinobacteria, obtained by whole-genome-amplification of dilution-to-extinction cultures. Sci. Rep. 7:42252 10.1038/srep42252 28186143 PMC5301498

[B61] Kato Y. Sato K. Asai K. Akutsu T. (2012). Rtips: fast and accurate tools for RNA 2D structure prediction using integer programming. Nucleic Acids Res. 40, 29–34. 10.1093/nar/gks.412 22600734 PMC3394313

[B62] Ke A. Doudna J. A. (2004). Crystallization of RNA and RNA? protein complexes. Methods 34, 408–414. 10.1016/j.ymeth.2004.03.027 15325657

[B63] Kim J. N. Breaker R. R. (2008). Purine sensing by riboswitches. Biol. Cell 100, 1–11. 10.1042/BC20070088 18072940

[B64] Klein D. J. (2006). Structural basis of glmS ribozyme activation by glucosamine-6-phosphate. Science 313, 1752–1756. 10.1126/science.1129666 16990543

[B65] Klein R. J. Eddy S. R. (2003). RSEARCH: finding homologs of single structured RNA sequences. BMC Bioinformatics 4:44. 10.1186/1471-2105-4-44 14499004 PMC239859

[B66] Krogh A. Brown M. Mian I. S. Sjölander K. Haussler D. (1994). Hidden Markov models in computational biology: applications to protein modeling. J. Mol. Biol. 235, 1501–1531. 10.1006/jmbi.1994.1104 8107089

[B67] Krokhotin A. Houlihan K. Dokholyan N. V. (2015). iFoldRNA v2: Folding RNA with constraints. Bioinformatics 31, 2891–2893. 10.1093/bioinformatics/btv221 25910700 PMC4547609

[B68] Kubodera T. Watanabe M. Yoshiuchi K. Yamashita N. Nishimura A. Nakai S.. (2003). Thiamine-regulated gene expression of *Aspergillus oryzae* thiA requires splicing of the intron containing a riboswitch-like domain in the 5′-UTR. FEBS Lett. 555, 516–520. 10.1016/S0014-5793(03)01335-8 14675766

[B69] Lai E. C. (2003). RNA sensors and riboswitches: self-regulating messages. Curr. Biol. 13, R285–R291. 10.1016/S0960-9822(03)00203-3 12676109

[B70] Lang K. Rieder R. Micura R. (2007). Ligand-induced folding of the thiM TPP riboswitch investigated by a structure-based fluorescence spectroscopic approach. Nucleic Acids Res. 35, 5370–5378. 10.1093/nar/gkm580 17693433 PMC2018614

[B71] Leyn S. A. Suvorova I. A. Kholina T. D. Sherstneva S. S. Novichkov P. S. Gelfand M. S.. (2014). Comparative genomics of transcriptional regulation of methionine metabolism in proteobacteria. PLoS ONE 9:e113714. 10.1371/journal.pone.0113714 25411846 PMC4239095

[B72] Li S. Breaker R. R. (2013). Eukaryotic TPP riboswitch regulation of alternative splicing involving long-distance base pairing. Nucleic Acids Res. 41, 3022–3031. 10.1093/nar/gkt057 23376932 PMC3597705

[B73] Li Y. Zhang C. Zhang S. (2012). Finding consensus stable local optimal structures for aligned RNA sequences, in 2nd IEEE International Conference on Computational Advances in Bio and Medical Sciences ICCABS 2012, Vol. 10 (Las Vegas, NV), 498–518.

[B74] Lorenz R. Bernhart S. H. Höner Zu Siederdissen C. Tafer H. Flamm C. Stadler P. F.. (2011). ViennaRNA Package 2.0. Algorithms Mol. Biol. 6:26. 10.1186/1748-7188-6-26 22115189 PMC3319429

[B75] Lu Z. J. Mathews D. H. (2008). Efficient siRNA selection using hybridization thermodynamics. Nucleic Acids Res. 36, 640–647. 10.1093/nar/gkm920 18073195 PMC2241856

[B76] Lu Z. J. Gloor J. W. Mathews D. H. (2009). Improved RNA secondary structure prediction by maximizing expected pair accuracy. RNA 15, 1805–1813. 10.1261/rna.1643609 19703939 PMC2743040

[B77] Lyskov S. Chou F. C. Conchúir S. Ó. Der B. S. Drew K. Kuroda D.. (2013). Serverification of molecular modeling applications: the rosetta online server that includes everyone (ROSIE). PLoS ONE 8:e63906. 10.1371/journal.pone.0063906 23717507 PMC3661552

[B78] Machtel P. Bąkowska-Żywicka K. Żywicki M. (2016). Emerging applications of riboswitches – from antibacterial targets to molecular tools. J. Appl. Genet. 57, 531–541. 10.1007/s13353-016-0341-x 27020791 PMC5061826

[B79] Magnus M. Boniecki M. J. Dawson W. Bujnicki J. M. (2016). SimRNAweb: a web server for RNA 3D structure modeling with optional restraints. Nucleic Acids Res. 44, W315–W319. 10.1093/nar/gkw279 27095203 PMC4987879

[B80] Magnus M. Matelska D. Lach G. Chojnowski G. Boniecki M. J. Purta E.. (2014). Computational modeling of RNA 3D structures, with the aid of experimental restraints. RNA Biol. 11, 522–536. 10.4161/rna.28826 24785264 PMC4152360

[B81] Mandal M. (2004). A glycine-dependent riboswitch that uses cooperative binding to control gene expression. Science 306, 275–279. 10.1126/science.1100829 15472076

[B82] Mandal M. Breaker R. R. (2004a). Adenine riboswitches and gene activation by disruption of a transcription terminator. Nat. Struct. Mol. Biol. 11, 29–35. 10.1038/nsmb710 14718920

[B83] Mandal M. Breaker R. R. (2004b). Gene regulation by riboswitches. Nat. Rev. Mol. Cell Biol. 5, 451–463. 10.1016/j.bpj.2012.10.026 15173824

[B84] Markham N. R. Zuker M. (2005). DINAMelt web server for nucleic acid melting prediction. Nucleic Acids Res. 33, 577–581. 10.1093/nar/gki591 15980540 PMC1160267

[B85] Markham N. R. Zuker M. (2008). UNAFold: software for nucleic acid folding and hybridization. Methods Mol. Biol. 453, 3–31. 10.1007/978-1-60327-429-6_1 18712296

[B86] Mathews D. H. Turner D. H. (2002). Dynalign: an algorithm for finding the secondary structure common to two RNA sequences. J. Mol. Biol. 317, 191–203. 10.1006/jmbi.2001.5351 11902836

[B87] Mathews D. H. Andre T. C. Kim J. Turner D. H. Zuker M. (1998). An updated recursive algorithm for RNA secondary structure prediction with improved thermodynamic parameters. Mol. Model. Nucleic Acids 682, 246–257. 10.1021/bk-1998-0682.ch015

[B88] Mathews D. H. Burkard M. E. Freier S. M. Wyatt J. R. Turner D. H. (1999a). Predicting oligonucleotide affinity to nucleic acid targets. RNA 5, 1458–1469. 10580474 10.1017/s1355838299991148 PMC1369867

[B89] Mathews D. H. Disney M. D. Childs J. L. Schroeder S. J. Zuker M. Turner D. H. (2004). Incorporating chemical modification constraints into a dynamic programming algorithm for prediction of RNA secondary structure. Proc. Natl. Acad. Sci. U.S.A. 101, 7287–7292. 10.1073/pnas.0401799101 15123812 PMC409911

[B90] Mathews D. H. Sabina J. Zuker M. Turner D. H. (1999b). Expanded sequence dependence of thermodynamic parameters improves prediction of RNA secondary structure. J. Mol. Biol. 288, 911–940. 10329189 10.1006/jmbi.1999.2700

[B91] Mattick J. S. (2001). Non-coding RNAs: the architects of eukaryotic complexity. EMBO Rep. 2, 986–991. 10.1093/embo-reports/kve230 11713189 PMC1084129

[B92] McCaskill J. S. (1990). The equilibrium partition function and base pair binding probabilities for RNA secondary structure. Biopolymers 29, 1105–1119. 10.1002/bip.360290621 1695107

[B93] Miao Z. Adamiak R. W. Antczak M. Batey R. T. Becka A. J. Biesiada M.. (2017). RNA-puzzles round III: 3D RNA structure prediction of five riboswitches and one ribozyme. RNA 23, 655–672. 10.1261/rna.060368.116 28138060 PMC5393176

[B94] Miao Z. Adamiak R. W. Blanchet M.-F. Boniecki M. Bujnicki J. M. Chen S.-J.. (2015). RNA-puzzles round II: assessment of RNA structure prediction programs applied to three large RNA structures. RNA 21, 1066–1084. 10.1261/rna.049502.114 25883046 PMC4436661

[B95] Mistry J. Finn R. D. Eddy S. R. Bateman A. Punta M. (2013). Challenges in homology search: HMMER3 and convergent evolution of coiled-coil regions. Nucleic Acids Res. 41:e121. 10.1093/nar/gkt263 23598997 PMC3695513

[B96] Moult J. Fidelis K. Kryshtafovych A. Schwede T. Tramontano A. (2014). Critical assessment of methods of protein structure prediction (CASP) - round x. Proteins Struct. Funct. Bioinforma. 82, 1–6. 10.1002/prot.24452 24344053 PMC4394854

[B97] Mukherjee S. Sengupta S. (2016). Riboswitch scanner: an efficient pHMM-based web-server to detect riboswitches in genomic sequences. Bioinformatics 32, 776–778. 10.1093/bioinformatics/btv640 26519506

[B98] Nahvi A. (2004). Coenzyme B12 riboswitches are widespread genetic control elements in prokaryotes. Nucleic Acids Res. 32, 143–150. 10.1093/nar/gkh167 14704351 PMC373277

[B99] Nawrocki E. P. (2014). Annotating functional RNAs in genomes using infernal Methods Mol. Biol. 1097, 163–197. 10.1007/978-1-62703-709-9_9 24639160

[B100] Nawrocki E. P. Eddy S. R. (2013a). Computational identification of functional RNA homologs in metagenomic data. RNA Biol. 10, 1170–1179. 10.4161/rna.25038 23722291 PMC3849165

[B101] Nawrocki E. P. Eddy S. R. (2013b). Infernal 1.1: 100-fold faster RNA homology searches. Bioinformatics 29, 2933–2935. 10.1093/bioinformatics/btt509 24008419 PMC3810854

[B102] Nawrocki E. P. Burge S. W. Bateman A. Daub J. Eberhardt R. Y. Eddy S. R.. (2015). Rfam 12.0: updates to the RNA families database. Nucleic Acids Res. 43, D130–D137. 10.1093/nar/gku1063 25392425 PMC4383904

[B103] Nelson J. W. Sudarsan N. Furukawa K. Weinberg Z. Wang J. X. Breaker R. R. (2013). Riboswitches in eubacteria sense the second messenger c-di-AMP. Nat. Chem. Biol. 9, 834–839. 10.1038/nchembio.1363 24141192 PMC3830699

[B104] Nudler E. Mironov A. S. (2004). The riboswitch control of bacterial metabolism. Trends Biochem. Sci. 29, 11–17. 10.1016/j.tibs.2003.11.004 14729327

[B105] Onoa B. Tinoco I. (2004). RNA folding and unfolding. Curr. Opin. Struct. Biol. 14, 374–379. 10.1016/j.sbi.2004.04.001 15193319

[B106] Ottink O. M. Rampersad S. M. Tessari M. Zaman G. J. R. Heus H. A. Wijmenga S. S. (2007). Ligand-induced folding of the guanine-sensing riboswitch is controlled by a combined predetermined induced fit mechanism. RNA 13, 2202–2212. 10.1261/rna.635307 17959930 PMC2080608

[B107] Pang E. Cao H. Zhang B. Lin K. (2015). Crop genome annotation: a case study for the brassica rapa genome, in Compendium of Plant Genomes, eds Wang X. Kole C. (Berlin; Heidelberg: Springer), 53–64.

[B108] Parisien M. Major F. (2008). The MC-Fold and MC-Sym pipeline infers RNA structure from sequence data. Nature 452, 51–55. 10.1038/nature06684 18322526

[B109] Peselis A. Serganov A. (2014). Themes and variations in riboswitch structure and function. Biochim. Biophys. Acta 1839, 908–918. 10.1016/j.bbagrm.2014.02.012 24583553 PMC4643838

[B110] Popenda M. Błazewicz M. Szachniuk M. Adamiak R. W. (2008). RNA FRABASE version 1.0: an engine with a database to search for the three-dimensional fragments within RNA structures. Nucleic Acids Res. 36, 386–391. 10.1093/nar/gkm786 17921499 PMC2238875

[B111] Popenda M. Szachniuk M. Antczak M. Purzycka K. J. Lukasiak P. Bartol N.. (2012). Automated 3D structure composition for large RNAs. Nucleic Acids Res. 40, 1–12. 10.1093/nar/gks339 22539264 PMC3413140

[B112] Popenda M. Szachniuk M. Blazewicz M. Wasik S. Burke E. K. Blazewicz J. (2010). RNA FRABASE 2. 0 : an advanced web-accessible database with the capacity to search the three-dimensional fragments within RNA structures. BMC Bioinformatics. 6:231 10.1186/1471-2105-11-231 PMC2873543 20459631

[B113] Purzycka K. J. Popenda M. Szachniuk M. Antczak M. Lukasiak P. Blazewicz J.. (2015). Automated 3D RNA structure prediction using the RNA composer method for riboswitches. Methods Enzymol. 553, 3–34. 10.1016/BS.MIE.2014.10.050 25726459

[B114] Qu Z. Adelson D. L. (2012). Evolutionary conservation and functional roles of ncRNA. Front. Genet. 3:205. 10.3389/fgene.2012.00205 23087702 PMC3466565

[B115] Ray P. S. Jia J. Yao P. Majumder M. Hatzoglou M. Fox P. L. (2009). A stress-responsive RNA switch regulates VEGFA expression. Nature 457, 915–919. 10.1038/nature07598 19098893 PMC2858559

[B116] Regulski E. E. Moy R. H. Weinberg Z. Barrick J. E. Yao Z. Ruzzo W. L.. (2008). A widespread riboswitch candidate that controls bacterial genes involved in molybdenum cofactor and tungsten cofactor metabolism. Mol. Microbiol. 68, 918–932. 10.1111/j.1365-2958.2008.06208.x 18363797 PMC2408646

[B117] Reining A. Nozinovic S. Schlepckow K. Buhr F. Fürtig B. Schwalbe H. (2013). Three-state mechanism couples ligand and temperature sensing in riboswitches. Nature 499, 355–359. 10.1038/nature12378 23842498

[B118] Remmele C. W. Xian Y. Albrecht M. Faulstich M. Fraunholz M. Heinrichs E.. (2014). Transcriptional landscape and essential genes of Neisseria gonorrhoeae. Nucleic Acids Res. 42, 10579–10595. 10.1093/nar/gku762 25143534 PMC4176332

[B119] Reuter J. S. Mathews D. H. (2010). RNAstructure: software for RNA secondary structure prediction and analysis. BMC Bioinformatics 11:129. 10.1186/1471-2105-11-129 20230624 PMC2984261

[B120] Rice G. M. Busan S. Karabiber F. Favorov O. V. Weeks K. M. (2014). SHAPE analysis of small RNAs and riboswitches. Methods Enzymol. 549, 165–187. 10.1016/B978-0-12-801122-5.00008-8 25432749 PMC4896901

[B121] Ritz J. Martin J. S. Laederach A. (2013). Evolutionary evidence for alternative structure in RNA sequence co-variation. PLoS Comput. Biol. 9:e1003152. 10.1371/journal.pcbi.1003152 23935473 PMC3723493

[B122] Roth A. Winkler W. C. Regulski E. E. Lee B. W. K. Lim J. Jona I.. (2007). A riboswitch selective for the queuosine precursor preQ1 contains an unusually small aptamer domain. Nat. Struct. Mol. Biol. 14, 308–317. 10.1038/nsmb1224 17384645

[B123] Rother M. Rother K. Puton T. Bujnicki J. M. (2011). ModeRNA: a tool for comparative modeling of RNA 3D structure. Nucleic Acids Res. 39, 4007–4022. 10.1093/nar/gkq1320 21300639 PMC3105415

[B124] Sato K. Kato Y. Hamada M. Akutsu T. Asai K. (2011). IPknot: fast and accurate prediction of RNA secondary structures with pseudoknots using integer programming. Bioinformatics 27, 85–93. 10.1093/bioinformatics/btr215 21685106 PMC3117384

[B125] Serganov A. Nudler E. (2013). A decade of riboswitches. Cell 152, 17–24. 10.1016/j.cell.2012.12.024 23332744 PMC4215550

[B126] Serganov A. Huang L. Patel D. J. (2008). Structural insights into amino acid binding and gene control by a lysine riboswitch. Nature 455, 1263–1267. 10.1038/nature07326 18784651 PMC3726722

[B127] Sharma S. Ding F. Dokholyan N. V. (2008). IFoldRNA: three-dimensional RNA structure prediction and folding. Bioinformatics 24, 1951–1952. 10.1093/bioinformatics/btn328 18579566 PMC2559968

[B128] Steger G. Hofmann H. Förtsch J. Gross H. J. Randies J. W. Sänger H. L.. (1984). Conformational transitions in viroids and virusoids: comparison of results from energy minimization algorithm and from experimental data. J. Biomol. Struct. Dyn. 2, 543–571. 10.1080/07391102.1984.10507591 6086063

[B129] Singh P. Bandyopadhyay P. Bhattacharya S. Krishnamachari A. Sengupta S. (2009). Riboswitch detection using profile hidden Markov models. BMC Bioinformatics 10:325. 10.1186/1471-2105-10-325 19814811 PMC2770071

[B130] Sloma M. F. Mathews D. H. (2015). Improving RNA secondary structure prediction with structure mapping data. Methods Enzymol. 553, 91–114. 10.1016/BS.MIE.2014.10.053 25726462

[B131] Smith C. Heyne S. Richter A. S. Will S. Backofen R. (2010). Freiburg RNA Tools: a web server integrating IntaRNA, ExpaRNA and LocARNA. Nucleic Acids Res. 38, 373–377. 10.1093/nar/gkq316 20444875 PMC2896085

[B132] Staple D. W. Butcher S. E. (2005). Pseudoknots: RNA structures with diverse functions. PLoS Biol. 3:e213. 10.1371/journal.pbio.0030213 15941360 PMC1149493

[B133] Sudarsan N. Lee E. R. Weinberg Z. Moy R. H. Kim J. N. Link K. H.. (2008). Riboswitches in eubacteria sense the second messenger cyclic Di-GMP. Science 321, 411–413. 10.1126/science.1159519 18635805 PMC5304454

[B134] Thore S. Leibundgut M. Ban N. (2006). Structure of the eukaryotic thiamine pyrophosphate riboswitch with its regulatory ligand. Science 312, 1208–1211. 10.1126/science.1128451 16675665

[B135] Tucker B. J. Breaker R. R. (2005). Riboswitches as versatile gene control elements. Curr. Opin. Struct. Biol. 15, 342–348. 10.1016/j.sbi.2005.05.003 15919195

[B136] Uzilov A. V. Keegan J. M. Mathews D. H. (2006). Detection of non-coding RNAs on the basis of predicted secondary structure formation free energy change. BMC Bioinformatics 7:173. 10.1186/1471-2105-7-173 16566836 PMC1570369

[B137] Vitreschak A. G. Rodionov D. A. Mironov A. A. Gelfand M. S. (2003). Regulation of the vitamin B12 metabolism and transport in bacteria by a conserved RNA structural element. RNA 9, 1084–1097. 10.1261/rna.5710303 12923257 PMC1370473

[B138] Wachter A. Tunc-Ozdemir M. Grove B. C. Green P. J. Shintani D. K. Breaker R. R. (2007). Riboswitch control of gene expression in plants by splicing and alternative 3′ end processing of mRNAs. Plant Cell 19, 3437–3450. 10.1105/tpc.107.053645 17993623 PMC2174889

[B139] Wacker A. Buck J. Mathieu D. Richter C. Wöhnert J. Schwalbe H. (2011). Structure and dynamics of the deoxyguanosine-sensing riboswitch studied by NMR-spectroscopy. Nucleic Acids Res. 39, 6802–6812. 10.1093/nar/gkr238 21576236 PMC3159443

[B140] Walter A. E. Turner D. H. Kim J. Lyttle M. H. Müller P. Mathews D. H.. (1994). Coaxial stacking of helixes enhances binding of oligoribonucleotides and improves predictions of RNA folding. Proc. Natl. Acad. Sci. U.S.A. 91, 9218–9222. 10.1073/pnas.91.20.9218 7524072 PMC44783

[B141] Wang J. X. Lee E. R. Morales D. R. Lim J. Breaker R. R. (2008). Riboswitches that Sense S-adenosylhomocysteine and activate genes involved in coenzyme recycling. Mol. Cell 29, 691–702. 10.1016/j.molcel.2008.01.012 18374645 PMC2712820

[B142] Wang J. Zhao Y. Zhu C. Xiao Y. (2015). 3dRNAscore: a distance and torsion angle dependent evaluation function of 3D RNA structures. Nucleic Acids Res. 43:e63. 10.1093/nar/gkv141 25712091 PMC4446410

[B143] Wheeler T. J. Eddy S. R. (2013). nhmmer: DNA homology search with profile HMMs. Bioinformatics 29, 2487–2489. 10.1093/bioinformatics/btt403 23842809 PMC3777106

[B144] Will S. Joshi T. Hofacker I. L. Stadler P. F. Backofen R. (2012). LocARNA-P: accurate boundary prediction and improved detection of structural RNAs. RNA 18, 900–914. 10.1261/rna.029041.111 22450757 PMC3334699

[B145] Will S. Reiche K. Hofacker I. L. Stadler P. F. Backofen R. (2007). Inferring noncoding RNA families and classes by means of genome-scale structure-based clustering. PLoS Comput. Biol. 3, 680–691. 10.1371/journal.pcbi.0030065 17432929 PMC1851984

[B146] Winkler W. Nahvi A. Breaker R. R. (2002). Thiamine derivatives bind messenger RNAs directly to regulate bacterial gene expression. Nature 419, 952–956. 10.1038/nature01145 12410317

[B147] Winkler W. C. Cohen-Chalamish S. Breaker R. R. (2002). An mRNA structure that controls gene expression by binding FMN. Proc. Natl. Acad. Sci. U.S.A. 99, 15908–15913. 10.1073/pnas.212628899 12456892 PMC138538

[B148] Winkler W. C. Nahvi A. Sudarsan N. Barrick J. E. Breaker R. R. (2003). An mRNA structure that controls gene expression by binding S-adenosylmethionine. Nat. Struct. Biol. 10, 701–707. 10.1038/nsb967 12910260

[B149] Xu X. Zhao P. Chen S. J. (2014). Vfold: a web server for RNA structure and folding thermodynamics prediction. PLoS ONE 9:e107504. 10.1371/journal.pone.0107504 25215508 PMC4162592

[B150] Zhao Y. Huang Y. Gong Z. Wang Y. Man J. Xiao Y. (2012). Automated and fast building of three-dimensional RNA structures. Sci. Rep. 2:734. 10.1038/srep00734 23071898 PMC3471093

[B151] Zuker M. (1989). On finding all suboptimal foldings of an RNA molecule. Science 244, 48–52. 10.1126/science.2468181 2468181

[B152] Zuker M. (2000). Calculating nucleic acid secondary structure. Curr. Opin. Struct. Biol. 10, 303–310. 10.1016/S0959-440X(00)00088-9 10851192

[B153] Zuker M. (2003). Mfold web server for nucleic acid folding and hybridization prediction. Nucleic Acids Res. 31, 3406–3415. 10.1093/nar/gkg595 12824337 PMC169194

[B154] Zuker M. Stiegler P. (1981). Optimal computer folding of large RNA sequences using thermodynamics and auxiliary information. Nucleic Acids Res. 9, 133–148. 10.1093/nar/9.1.133 6163133 PMC326673

